# Physical, Morphological, and Rheological Properties of Agglomerated Milk Protein Isolate Powders: Effect of Binder Type and Concentration

**DOI:** 10.3390/polym15020411

**Published:** 2023-01-12

**Authors:** Yulim Jeong, Byoungseung Yoo

**Affiliations:** Department of Food Science and Biotechnology, Dongguk University-Seoul, Goyang 410-820, Republic of Korea

**Keywords:** milk protein isolate powder, sugar binder, fluidized-bed agglomeration, physical property, rheological property

## Abstract

Milk protein isolate powder (MPIP), a high protein-based powder, is a common dietary ingredient but has poor physical properties due to its cohesive nature. Powder agglomeration is one of the most widely used methods to improve and modify the quality of MPIP structures. In this study, the physical, morphological, and rheological properties of MPIPs agglomerated in a fluidized-bed agglomeration process were investigated as a function of sugar binder type and concentration. The physical properties of MPIP were evaluated by their flowability, cohesiveness, porosity, particle size distribution (PSD), and water-holding properties (wettability, solubility, and water-binding capacity). The density values of the agglomerated MPIPs decreased with increasing the binder concentration, whereas the porosity, wettability, and solubility values increased. Such trends were consistent with SEM observations. The MPIP agglomerated with 10% sorbitol had the largest particle diameter (D_50_) and showed better physical properties compared to the other sugar binders. The viscosity values (η_a,50_) of the MPIPs agglomerated with sugar binders showed lower values than the control (no sugar binder). The agglomeration process enhanced the viscoelasticity of the MPIP, but the viscoelasticity decreased with increasing the sugar binder concentration. These observations suggested that the physical, morphological, and rheological properties of MPIP can be greatly affected by the binder type and concentration in the agglomeration process.

## 1. Introduction

Milk protein exhibits a variety of properties, such as high nutrition, easy digestion, GRAS status (‘generally regarded as safe’), and good compatibility with other components in food compositions [1]. Therefore, the use of milk protein is growing rapidly. As a powder, milk protein plays an important role in the food industry because of its convenience for processing, preservation, and transportation [2]. Milk protein isolate powder (MPIP) is commonly used to make cheese, drinks, yogurt, and other meals [3] and to improve the texture of food products. MPIP is usually produced by ultrafiltration of skim milk to remove lactose and minerals and is then dehydrated by spray drying, keeping the casein-to-whey protein ratio close to that of raw milk [4,5]. However, due to its cohesive nature, such a high-protein dairy product is difficult to utilize because of its poor flowability and wettability [6,7]. Powder agglomeration is one of the most widely used methods to solve these problems.

Agglomeration is a size-enlargement process that results in a porous aggregate that is larger than the original particles [8,9]. In general, spray dryers, extruders, and fluidized beds have been commonly used in the food industry as powder agglomeration equipment. Using a binder solution in the fluidized-bed agglomeration process (FBAP) is one of the most common ways to change the structure of agglomerates. The FBAP consists of three steps: wetting a liquid binder on dry powder and nucleating primary particles, compression to combine the nucleus and agglomerates, and agitation to break up agglomeration and attrition [8]. It is known that changing the structures of food biopolymer powders enhances physical properties (flowability, cohesiveness, dispersibility, wettability, and solubility), morphological properties (size, density, surface, and porosity), and rheological properties [8,9,10]. Major applications of FBAP in the food industry include dispersible powdered milk and soup mixtures, instant chocolate mixtures, beverage powders, and food thickeners [11]. Due to differences in chemical compositions, mechanical qualities, concentration, viscosity, and inter-particle interactions between the particles and the binders, each sugar binder has a distinct bonding efficiency [12]. It is well-known that the structural characteristics of agglomerated powders are greatly affected by the type and concentration of the binder [11,12,13]. Recently, Lee et al. [8] and Lee and Yoo [11] applied the same concentration of various sugar binders to gum powders. They found that the addition of sugar binders in the FBAP greatly enhanced the flow characteristics and physical properties of the powders. When using the FBAP to produce the required and desirable powder product, it is critical to understand the association between the concentration of the sugar binder and the structural and physical qualities. Structural modifications of powders agglomerated with different sugar concentrations are especially important because they alter the powder’s flowability, wettability, and solubility.

In general, the physical properties of protein powder affect its handling and processing. For MPIP, the water-holding properties (wettability, water-binding capacity, and solubility) are particularly important because the powder is dissolved in a liquid before use [14]. Furthermore, a thorough and rapid dispersion of milk protein powder at room temperature is essential in many food applications [15]. Fast wetting is favored by large particles of high porosity and small contact angles [1]. Solubility is another important functional property of MPIP as it affects other functional properties, such as foaming, gelling, and emulsifying [15]. Therefore, understanding the factors that affect the physical properties of MPIP could provide useful insights when applying MPIP in food systems.

Despite the importance of using sugar binders in the agglomeration process of MPIP, there have been no investigations yet about the influence of the type and concentration of different sugar binders on the physical, structural, and rheological properties of MPIP agglomerated by FBAP. Therefore, in this study, MPIP was agglomerated with four different sugar binders (sucrose, lactose, xylitol, and sorbitol) at different concentrations (0%, 10%, and 20%), and the main objective of this study was to investigate the effect of sugar binders with different concentrations on the physical and structural, and rheological properties of agglomerated MPIPs prepared by FBAP.

## 2. Materials and Methods

### 2.1. Materials

MPIP (Tricom Trade Co., Ltd., Seoul, Republic of Korea), with 81.5% protein, 8% ash, 4% carbohydrate, 5% moisture, and 1.5% fat, was used in this study to produce agglomerates. The following sugars were used for the binder solutions: sucrose (Samyang Co., Ltd., Seongnam, Republic of Korea), lactose (Meggle Co., Ltd., Wasserburg, Germany), xylitol (Crown Co., Jincheon, Republic of Korea), and sorbitol (Samyang Co., Ltd.).

### 2.2. Fluidized-Bed Agglomeration Process (FBAP)

MPIP was agglomerated using a top-spray fluidized-bed granulator (Fluid Bed Lab System, Dae Ho Technology Co., Ltd., Hwaseong, Republic of Korea). Binder solutions were prepared by completely dissolving different sugars in distilled water at concentrations of 0%, 10%, and 20% (*w*/*w*) [11,16]. To ensure complete hydration, each solution was agitated with a mechanical mixer for 20 min before being kept at room temperature overnight. MPIP (1000 g) was added to the chamber and fluidized by an upward-flowing air stream. The following agglomeration conditions were used: flow rate of the binder solution: 16.65 mL/min; product temperature: 53 ± 1 °C; spray pressure: 1.5 bar; blower: 80%; damper: 30%. All samples were dried and cooled in the chamber until the agglomerated powder was 40 °C after the spraying process.

### 2.3. Particle Size Distribution (PSD)

Volume-based PSD measurements were obtained using a laser diffraction particle size analyzer (Mastersizer 3000E, Malvern Instruments Ltd., Worcestershire, UK). The D_10_, D_50_, and D_90_ values were the corresponding particle diameters at 10%, 50%, and 90% in the cumulative size distribution. The span value was calculated by the following equation:

(1) $$\mathrm{Span}=\frac{D_{90}-D_{10}}{D_{500}}$$

### 2.4. Flowability, Cohesiveness, and Porosity Measurements

The bulk density (*ρ*_bulk_) of the samples was obtained from the ratio of mass to aerated bulk volume by measuring the volume after adding a powder of constant weight in a 100 mL cylinder. The tapped density (*ρ*_tapped_) was calculated by measuring the ratio of mass and tapped volume of the samples after tapping 1250 times with a tap density volumeter (BT-301, K-ONE Ltd., Seoul, Republic of Korea). The Carr index (CI) and Hausner ratio (HR) were determined from the *ρ*_tapped_ and *ρ*_bulk_ of the powder by the following equations:

(2) $$\mathrm{CI}=\frac{\rho_{\mathrm{tapped}}-\rho_{\mathrm{bulk}}}{\rho_{\mathrm{tapped}}}\times 100$$

Flowability (%) of powders according to CI was classified as follows: <15: very good; 15–20: good; 20–35: fair; 35–45: bad; >45: very bad [17].

(3) $$\mathrm{HR}=\frac{\rho_{\mathrm{tapped}}}{\rho_{\mathrm{bulk}}}$$

The cohesiveness of the powder was classified as follows: <1.2: free-flowing; 1.2–1.4: intermediate flowing; >1.4: highly cohesive and non-flowing [18].

To determine the porosity of the powder, which indicates the void of the powder, 0.5 g of MPIP was put into a 10 mL cylinder followed by the addition of 5 mL of petroleum ether to fill the porous spaces of the powder, and then 1 mL of petroleum ether was added to clear the cylinder’s wall. Particle density (*ρ*_particle_) and porosity (ε) were calculated by the following equations:

*ρ*_particle_ = W_p_/(V_t_ − 6) (4)

where W_p_ is the weight of the powder (g), and V_t_ is the total volume of petroleum ether and suspended powder (mL).

ε = (*ρ*_particle_ − *ρ*_tapped_)/*ρ*_particle_ (5)

### 2.5. Water-Binding Capacity Measurement

According to the procedure provided by Mirmoghtadaie et al. [19], 1 g of sample dispersed in 10 mL of distilled water was transferred to a pre-weighed centrifuge tube. The solution was then vortexed for 10 s and centrifuged using a centrifugal separator (CT 6E, Hitachi Co., Ltd., Hitachi, Japan) at 700× *g* for 10 min. The supernatant was decanted, and the tube was reweighed. Water-binding capacity was expressed as grams of water per gram of protein.

### 2.6. Wettability Measurement

The Washburn method [20] was used to determine wettability by using the capillary force to wet the powder. To this end, 2 g of sample was added to an open-ended glass tube covered with filter paper at the bottom. Then, the tube was put just above the distilled water (24 °C) surface. After 10 min, the mass of the wetted powder was measured. Wettability was calculated as the weight of the water absorbed by MPIP.

### 2.7. Solubility Measurement

Solubility measurements were determined by the percentage suspension stability (%SS) method introduced by Sikand et al. [15]. Aqueous solutions of MPIPs (5%, *w*/*w*) were completely dispersed by blending the MPIP with distilled water for 30 min. The solution was centrifuged using a centrifugal separator (CT 6E, Hitachi Co., Ltd.) at 700× *g* for 10 min. The supernatant was pipetted into a pre-weighed beaker, which was then dried overnight in a dry oven at 105 °C for complete evaporation and was then reweighed. The total solid (TS) content of the samples was calculated by the following equation:

(6) $$\%\mathrm{SS}=\frac{A}{B}\times 100$$

where *A* is the TS content of the supernatant, and *B* is the TS content of the original dispersion.

### 2.8. Scanning Electron Microscopy (SEM)

MPIP was attached to an aluminum fragment using double-sided adhesive carbon tape and coated with platinum–palladium in a vacuum. Particle morphology was identified using an SEM (Hitachi S-3000 N, Hitachi Co., Ltd.) operating at 20 kV and a magnification of 150×.

### 2.9. Sample Preparation for Rheological Properties

Before the rheological measurements, the agglomerated and raw MPIPs were dispersed in distilled water to obtain solutions of 15% (*w*/*w*) protein concentration. After dispersion, the solutions were maintained at 4 °C overnight to fully hydrate the agglomerated and raw MPIPs.

### 2.10. Rheological Properties

Flow and dynamic rheological measurements were conducted using a controlled stress rheometer (Haake RheoStress 1, Haake GmbH, Karlsruhe, Germany) at 25 °C. In both measurements, the plate–plate geometry was used, the plate diameter was 35 mm, and the gap between the plates was set to 500 µm. To investigate the flow behavior of the solution, shear stress was measured at a shear rate range of 0.1–100 s^−1^. The results were entered into the power law equation below to calculate the consistency index (K) and flow behavior index (n):

(7) $$\sigma =K\cdot {\dot{\gamma }}^{n}$$

where σ is the shear stress (Pa), and $\dot{\gamma }$ (s^−1^) is the shear rate.

Dynamic viscoelastic properties were measured using small amplitude oscillatory rheological measurements at 25 °C. A dynamic oscillatory test was determined in the angular frequency range of 0.63–62.8 rad·s^−1^ with 2% strain within the linear viscoelasticity limit. From the measurement, the storage modulus (G′), loss modulus (G″), and loss tangent (tan δ; G″/G′) of the solution at 6.28 rad·s^−1^ were calculated by Haake Rheowin software (ver. 4.50.0003; Haake GmbH, Haren, Germany).

### 2.11. Statistical Analysis

For all tests, three measurements were conducted. Experimental data (mean ± standard deviation) were subjected to analysis of variance (ANOVA) using the SAS software version 9.4 (SAS Institute, Cary, Inc., Farmingdale, NY, USA). Duncan’s multiple range test was applied to determine the difference among means at a statistical significance of *p* < 0.05.

## 3. Results

### 3.1. PSD Analysis

**Figure 1:**
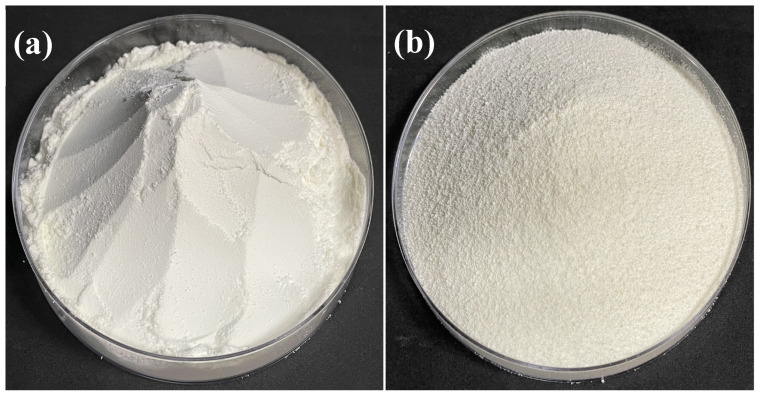
Typical sample appearance of (**a**) raw and (**b**) agglomerated MPIPs prepared by FBAP.

**Figure 2:**
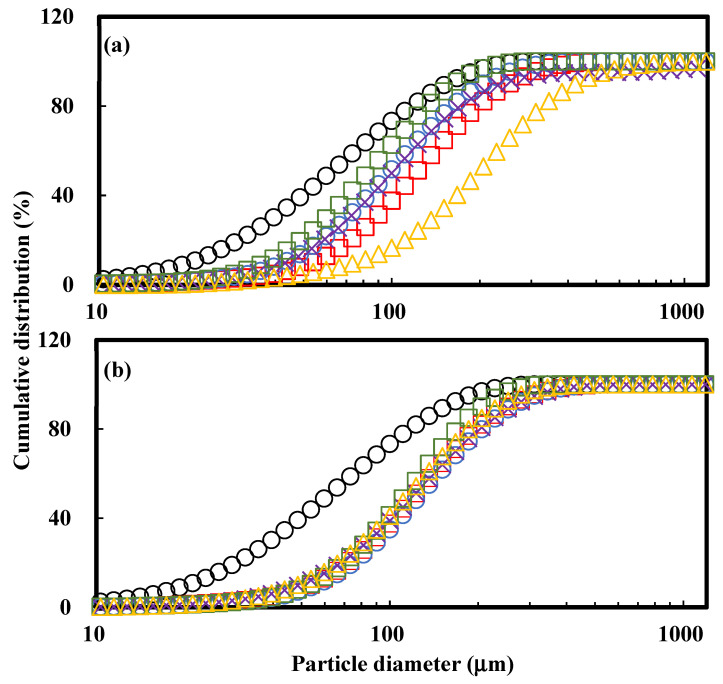
Cumulative distribution of raw (○) and agglomerated MPIPs with various sugar binders at (**a**) 10% and (**b**) 20% binder concentrations: control (no sugar) (□), sucrose (○), lactose (×), xylitol (□), and sorbitol (△).

Agglomeration is the process of binding small particles into large particles. Such changes in the particle size affect the other physical properties of the powder, so it is an important characteristic when using powder in the food industry. As can be seen with the naked eye, the agglomerated MPIP (10% sorbitol) (Figure 1b) prepared by FBAP had larger particles than the raw MPIP, indicating that the FBAP greatly increased the size of the MPIP (Figure 1a). Table 1 and Figure 2 present the particle diameters (D_10_, D_50_, and D_90_) of the raw and agglomerated MPIPs prepared with various sugar binders at different concentrations. The D_50_ of all the agglomerates, except those agglomerated with sorbitol, increased with the concentration of the binder. Increasing the concentration of a liquid binder decreases the inter-particle void space, such that the gaps among the particles are saturated with liquid, promoting particle growth [20]. In addition, it is well-known that the particle size increases when sugar binders are applied to powdered foods [11,21,22]. For the 10% binders, the particle size increased in the order of xylitol < sucrose < lactose < sorbitol, and for the 20% binders, the particle size increased in the order of xylitol = sorbitol < lactose < sucrose, indicating that the particle size can be affected by the type of sugar binder.

The span value, which indicates the width of the PSD, often serves as the foundation for evaluating the homogeneity of agglomerates and their dispersity [23]. The range of the span value of the MPIP agglomerated with four sugar binders was 1.36–1.87, which was much lower than that of the raw MPIP (2.15). This indicated that the FBAP increased the homogeneity of the powders. The sample with the largest span value (1.87) was the MPIP agglomerated with 10% sorbitol, which seemed to be because it had the largest particle size and thus was easy to break and had more fine particles. In addition, for the samples agglomerated with all sugar binders, the higher the concentration of the binder, the lower the span value, which can be attributed to the lower friability [24]. One of the purposes of agglomeration is to achieve a consistent-sized powder [25]. Therefore, it was found that as the sugar binder concentration increased, more uniform agglomerates could be obtained.

### 3.2. Flowability, Cohesiveness, and Porosity (ε)

The CI, HR, and porosity (ε) of the raw and agglomerated MPIPs with various sugar binders at different concentrations are shown in Table 2. The CI (17.0–21.7) and HR (1.19–1.27) values of the agglomerated samples were significantly lower than the CI (26.7) and HR (1.36) of the raw sample. In addition, all agglomerated samples showed fair or good flowability, with intermediate cohesiveness or free-flowing properties. This implied that the FBAP affected the flowability and cohesiveness, and the flowability and cohesiveness of the MPIP samples were also improved when using sugar binders in the FBAP. The sample agglomerated with 10% sorbitol had the lowest CI and HR values among the samples agglomerated with sugar binders, indicating better flowability and lower cohesiveness. This result also indicated that the size enlargement caused by the FBAP significantly influenced the flowability and cohesiveness of the MPIPs. The *ρ*_bulk_ and *ρ*_tapped_ of the agglomerated samples were lower than those of the raw sample, and as the binder concentration increased, the *ρ*_bulk_ and *ρ*_tapped_ values decreased. A porous structure forms during the FBAP, which involves occluded air or vacuoles, and thus the density of the raw powder was typically higher than the agglomerated powder [25].

The ε-value, or void fraction, of a powder sample is crucial to the internal microstructure of agglomerated powders. Table 2 shows that the ε-values of the agglomerated MPIPs (76.9–86.5) were higher compared to the raw MPIP (65.5) due to a particle-clustering phenomenon in the FBAP. In addition, all the samples agglomerated with sugar binders had an increased ε-value compared to the control (0% sugar), and, except for sorbitol, the ε-values increased with increasing the concentration of the sugar binder. These findings were consistent with previous research by Lee and Yoo [26], which suggested that the development of large globular clusters may be caused by the large droplet size in the existence of binders at high concentrations. However, with sorbitol, the ε-value decreased as the binder concentration increased because the MPIP agglomerated with a 10% sorbitol had a large particle size compared to the other samples (Table 1).

### 3.3. Water-Holding Properties

Table 3 shows the results for the water-holding properties of the raw and agglomerated MPIPs with various sugar binders at different concentrations. For various functional uses of proteins, solubility is a crucial prerequisite and a major functional feature. All the agglomerated samples showed higher solubility values (35.7–49.2) than the raw sample (31.1). It is well-known that the presence of small particles leads to poor instantiation properties and impaired water penetration [27]. The increased solubility of the agglomerated samples compared to the control indicated that agglomeration with sugar binders positively influenced the solubility. In addition, except for sorbitol, solubility increased as the concentration of the binder increased. This was because the increased sugar concentration facilitated faster moisture absorption [21].

Wettability is another important property of powders and agglomerates. It refers to the ability of a powder to penetrate the surface of still water and absorb water. When a powder is wet, water fills the voids. It mostly depends on the powder’s particle size, density, porosity, surface charge, surface area, the presence of amphipathic substances, and the surface activity of the particles [28]. In the present study, the agglomerated samples showed higher wettability than the raw sample. It is known that high-protein powder forms a hydrophobic layer that is impermeable and keeps water and powders apart, making it difficult to absorb water into the powder [5]. However, the wettability of MPIP can be expected to be improved by the addition of the sugar binder because the sugar contains hydrophilic groups, which makes it easier to wet by forming a hydrophilic bridge [14]. The fact that the MPIP agglomerated with 10% sorbitol, which had the largest particle diameter, displayed the best wettability was also consistent with the results of a previous study conducted by Ji et al. [14]. Large particles can enable the formation of large porous structures and the penetration of water through these structures [29]. Further research is needed, as the wettability of MPIP varies depending on the particle size and binder type. 

The agglomerated samples showed a larger water-binding capacity (2.39–3.93) than the raw sample. In addition, it can be shown that the water-binding capacity increased with an increase in porosity because the larger the spaces between the particles, the easier the water penetrates the powder. The MPIPs agglomerated with sugar binders showed higher water-binding capacities than the agglomerates without sugar, demonstrating that the FBAP with sugar binders positively affected the water-binding capacity.

### 3.4. Morphology

**Figure 3:**
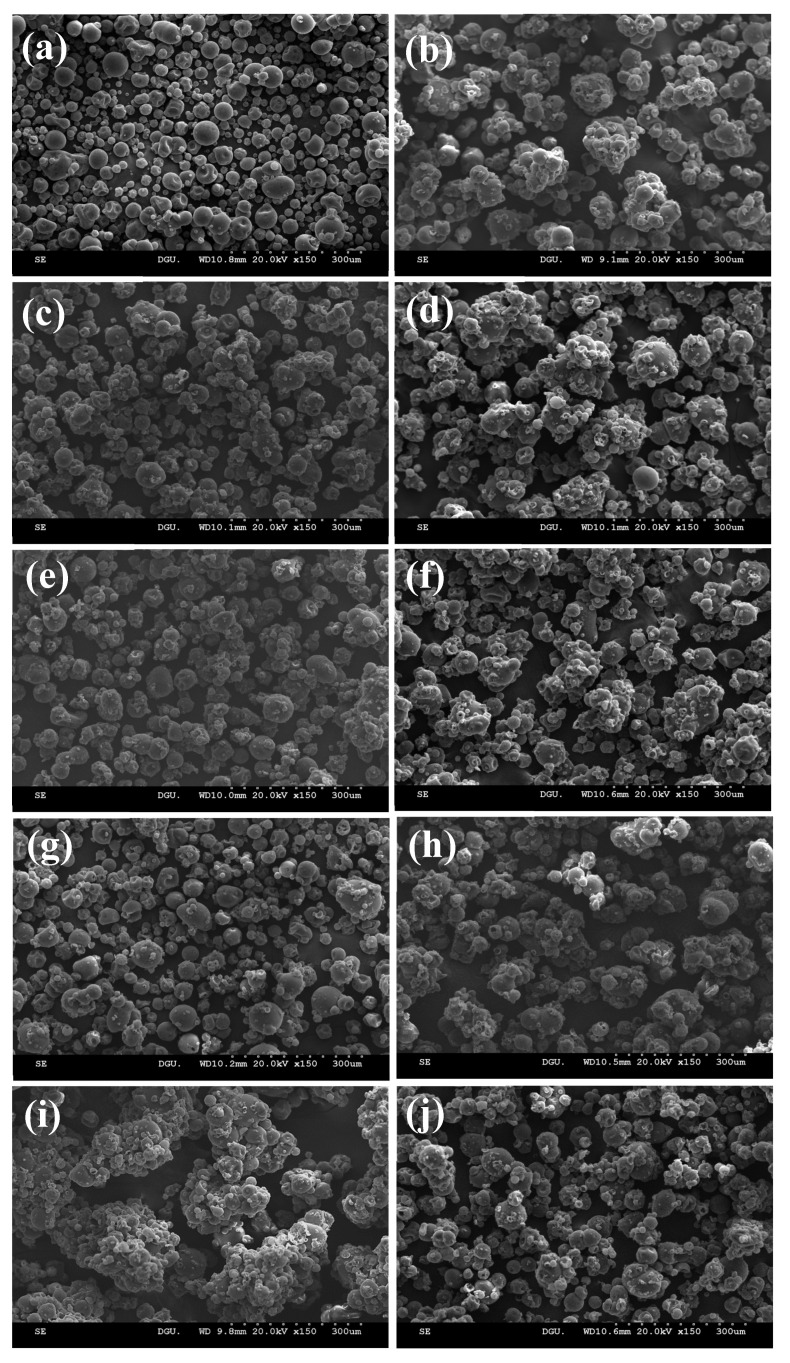
SEM micrographs of (**a**) raw and (**b**–**j**) agglomerated MPIPs with various sugar binders at different concentrations: (**b**) control (no sugar), (**c**) sucrose-10%, (**d**) sucrose-20%, (**e**) lactose-10%, (**f**) lactose-20%, (**g**) xylitol-10%, (**h**) xylitol-20%, (**i**) sorbitol-10%, (**j**) sorbitol-20%. Magnification 150×.

SEM was used to determine the gross morphology of the raw MPIP (Figure 3a) and the MPIPs (Figure 3b–j) agglomerated with various sugar binders at different concentrations. The agglomerated MPIPs revealed large, irregular-shaped particles with porous structures because of the smaller particles sticking together. By contrast, the raw MPIP showed a small, compact, spherical, and smooth surface. Agglomerated food powders are known to have a range of sizes, shapes, and structures [30]. The particle sizes of the agglomerated MPIPs were much larger than the raw MPIP, and their size increased with increasing the binder concentration from 10% to 20%, except for the MPIP agglomerated with sorbitol. Among the samples, the MPIP agglomerated with 10% sorbitol had the largest particle size and the most porous structure.

### 3.5. Rheological Properties

Table 4 shows the results of the power law model parameters of 15% (*w*/*w*) dispersion solutions of the raw and agglomerated MPIPs with various sugar binders at different concentrations. The experimental results of shear stress (*σ*) versus shear rate ($\dot{\gamma }$) were well-fitted to the power law model with a high *R*^2^ value (0.99). For all the samples, the *K*-value decreased as the binder concentration increased. Therefore, the sugar binder negatively affected the viscosity. This was because the sugars agglomerated with the MPIP, which decreased the total amount of protein in the MPIP agglomerates. All the samples had a flow behavior index (*n* = 0.39–0.59) lower than 1, indicating a shear-thinning behavior. That is, as the shear rate increased, the viscosity decreased. The *n* value of the MPIPs agglomerated with sugar increased as the binder concentration was increased from 10% to 20%, indicating a reduction in the pseudoplasticity with increasing the binder concentration. Compared to the control, the *K* values of the MPIPs agglomerated with 10% sugar were higher, whereas those with 20% sugar were lower, indicating that the viscosity of MPIP was higher at lower sugar binder concentrations.

Table 5 shows the dynamic viscoelastic characteristics of the raw and agglomerated MPIPs with various sugar binders at different concentrations. Viscoelasticity is a crucial property of high-protein concentration solutions that can provide vital details about intermolecular interactions [31]. The *G*′ and *G*′′ values, representing the elastic and viscous properties at 6.28 rad·s^−1^, were higher in all the agglomerated samples than in the raw sample. The tan *δ* values were also lower in the agglomerated MPIPs than in the raw sample. However, as the binder concentration increased from 10% to 20%, the *G*′ and *G*″ values decreased. This indicated that there may have been structural changes that caused variances in the molecular interaction behavior between the sugar binders and protein powders [32].

For all the samples, except those prepared with sugar alcohols (xylitol and sorbitol), *G*′′ was higher than *G*′, reflecting that the viscous property predominated in the MPIPs agglomerated with lactose or sucrose. In addition, tan *δ* increased as the binder concentration increased, showing that the sugar concentration had a pronounced effect on the viscous property of the sample. The MPIPs with 10% xylitol and 10% sorbitol showed relatively higher dynamic moduli values than the other agglomerated MPIPs, which was probably due to the better intermolecular interaction between sugar and milk protein. Their tan *δ* values (10% xylitol = 0.92; 10% sorbitol = 0.96) were also lower, indicating better intermolecular interaction. These findings illustrated that in the FBAP, the type of sugar binder and the concentration of the sugar binder both significantly influenced the steady and dynamic rheological properties of the agglomerated MPIPs. Further studies on other food proteins agglomerated in the FBAP are needed to expand on the results of this study. 

## 4. Conclusions

The physical, morphological, and rheological properties of MPIPs agglomerated with various sugar binders at different concentrations by the FBAP were evaluated and compared. All of the powders were prepared under the same agglomeration conditions. The flowability and cohesiveness of MPIP were improved by increasing the particle size of the powder due to the FBAP. The results indicated that the use of sugar binder increased the porosity of the MPIPs by the FBAP and had a pronounced impact on the water-holding properties of the MPIPs due to the enlargement of the particle size. In addition, as the binder concentration increased, the water-holding property values increased, indicating that the binder concentration greatly affected the physical properties of the MPIPs. Among the different concentrations and types of sugar binders, the agglomeration with 10% sorbitol produced the largest particle size and the most porous structure with improved flowability, cohesiveness, and water-holding properties. From the results of the rheological characteristics, it can be predicted that the viscosity decreases as the binder concentration increases due to the decrease in the total amount of protein per particle. The dynamic rheological measurements revealed that agglomeration with the sugar binder increased the viscoelasticity of the MPIPs, but as the binder concentration increased, the viscoelasticity decreased. In general, the agglomerated MPIPs with sorbitol seemed more efficient in improving the viscoelastic properties with relatively higher dynamic moduli; moreover, they had lower tan *δ* values. Therefore, the sugar binder type and concentration greatly affected the physical, morphological, and rheological properties of the agglomerated MPIPs. Specific research on the physical, morphological, and rheological properties of agglomerated MPIPs would be helpful for selecting the optimum sugar binder in the FBAP.

## Figures and Tables

**Table 1:** Particle diameters of raw and agglomerated MPIPs prepared with various sugar binders at different concentrations.

Sample	Binder Type	Binder Conc. (%)	D_10_ (mm)	D_50_ (mm)	D_90_ (mm)	Span
Raw MPIP			20.4 ± 0.01 ^i^	61.4 ± 0.09 ^g^	152 ± 1.93 ^g^	2.15 ± 0.03 ^a^
Agglomerated MPIP	Control (no sugar)	0	53.5 ± 0.14 ^c^	122 ± 0.35 ^c^	254 ± 0.73 ^c^	1.65 ± 0.01 ^e^
	Sucrose	10	42.7 ± 0.64 ^g^	97.4 ± 1.78 ^e^	205 ± 5.40 ^e^	1.66 ± 0.00 ^e^
		20	55.9 ± 0.11 ^b^	126 ± 0.20 ^b^	261 ± 0.86 ^b^	1.63 ± 0.01 ^f^
	Lactose	10	43.6 ± 0.05 ^f^	99.5 ± 0.29 ^e^	230 ± 5.42 ^d^	1.84 ± 0.01 ^c^
		20	48.7 ± 0.35 ^d^	122 ± 1.72 ^c^	260 ± 2.19 ^b^	1.74 ± 0.01 ^d^
	Xylitol	10	36.9 ± 0.02 ^h^	82.3 ± 0.19 ^f^	164 ± 0.33 ^f^	1.54 ± 0.01 ^g^
		20	52.8 ± 0.06 ^c^	112 ± 0.32 ^d^	206 ± 0.30 ^e^	1.36 ± 0.01 ^h^
	Sorbitol	10	73.6 ± 0.04 ^a^	192 ± 2.74 ^a^	432 ± 0.10 ^a^	1.88 ± 0.01 ^b^
		20	47.7 ± 0.25 ^e^	112 ± 1.82 ^d^	232 ± 3.42 ^d^	1.62 ± 0.01 ^f^

The values represent the means of triplicate measurements ± SD. Means with different lowercase letters ^a–i^ within each column at each binder type are significantly different (*p* < 0.05).

**Table 2:** Flow characteristics and porosity of raw and agglomerated MPIPs prepared with various sugar binders at different concentrations.

Sample	Binder Type	Binder Conc. (%)	*ρ*_bulk_ (g/cm^3^)	*ρ*_tapped_ (g/cm^3^)	ε (%)	CI (%)	HR
Raw MPIP			0.32 ± 0.00 ^a^	0.43 ± 0.00 ^a^	65.5 ± 0.00 ^i^	26.7 ± 0.29 ^a^	1.36 ± 0.01 ^a^
Agglomerated MPIP	Control (no sugar)	0	0.31 ± 0.00 ^b^	0.39 ± 0.01 ^c^	76.9 ± 0.00 ^h^	20.3 ± 0.58 ^b^	1.26 ± 0.01 ^c^
	Sucrose	10	0.31 ± 0.00 ^b^	0.38 ± 0.00 ^cd^	77.1 ± 0.00 ^g^	20.0 ± 0.00 ^c^	1.25 ± 0.00 ^d^
		20	0.28 ± 0.00 ^e^	0.35 ± 0.00 ^e^	79.1 ± 0.00 ^e^	20.0 ± 0.00 ^c^	1.25 ± 0.00 ^d^
	Lactose	10	0.30 ± 0.01 ^c^	0.38 ± 0.00 ^d^	77.1 ± 0.00 ^g^	21.0 ± 0.82 ^b^	1.27 ± 0.02 ^bc^
		20	0.28 ± 0.00 ^e^	0.35 ± 0.00 ^e^	78.2 ± 0.00 ^f^	21.7 ± 0.58 ^b^	1.28 ± 0.01 ^b^
	Xylitol	10	0.32 ± 0.00 ^a^	0.40 ± 0.00 ^b^	84.0 ± 0.00 ^c^	20.0 ± 0.00 ^c^	1.24 ± 0.01 ^e^
		20	0.29 ± 0.00 ^d^	0.35 ± 0.00 ^e^	86.0 ± 0.00 ^b^	17.0 ± 0.00 ^e^	1.20 ± 0.00 ^f^
	Sorbitol	10	0.28 ± 0.00 ^e^	0.34 ± 0.00 ^f^	86.5 ± 0.00 ^a^	15.0 ± 0.00 ^f^	1.19 ± 0.01 ^g^
		20	0.26 ± 0.00 ^f^	0.32 ± 0.00 ^g^	80.6 ± 0.00 ^d^	19.5 ± 0.41 ^d^	1.24 ± 0.01 ^e^

The values represent the means of triplicate measurements ± SD. Means with different lowercase letters ^a–i^ within each column at each binder type are significantly different (*p* < 0.05).

**Table 3:** Water-holding properties of raw and agglomerated MPIPs with various sugar binders at different concentrations.

Sample	Binder Type	Binder Conc. (%)	Solubility (%)	Wettability	Water-Binding Capacity (g/g)
Raw MPIP			31.1 ± 0.50 ^i^	0.97 ± 0.03 ^h^	2.11 ± 0.11 ^i^
Agglomerated MPIP	Control (no sugar)	0	34.5 ± 0.23 ^h^	1.29 ± 0.04 ^c^	2.39 ± 0.07 ^h^
	Sucrose	10	35.7 ± 0.12 ^h^	1.04 ± 0.03 ^g^	2.66 ± 0.01 ^g^
		20	39.6 ± 0.20 ^d^	1.21 ± 0.02 ^e^	3.01 ± 0.11 ^e^
	Lactose	10	36.5 ± 0.50 ^g^	1.13 ± 0.03 ^f^	2.56 ± 0.13 ^h^
		20	39.1 ± 0.23 ^e^	1.25 ± 0.01 ^d^	2.96 ± 0.04 ^f^
	Xylitol	10	37.7 ± 0.12 ^f^	1.06 ± 0.01 ^g^	3.32 ± 0.03 ^c^
		20	45.7 ± 0.81 ^b^	1.20 ± 0.01 ^e^	3.53 ± 0.08 ^b^
	Sorbitol	10	49.2 ± 0.20 ^a^	1.76 ± 0.07 ^a^	3.93 ± 0.18 ^a^
		20	41.3 ± 0.46 ^c^	1.47 ± 0.04 ^b^	3.19 ± 0.04 ^d^

The values represent the means of triplicate measurements ± SD. Means with different lowercase letters ^a–i^ within each column at each binder type are significantly different (*p* < 0.05).

**Table 4:** Values of power law model parameters of raw and agglomerated MPIPs prepared with various sugar binders at various concentrations.

Sample	Binder Type	Binder Conc. (%)	K (Pa·s^n^)	n	R^2^
Raw MPIP			1.33 ± 0.01 ^d^	0.55 ± 0.01 ^c^	0.99
Agglomerated MPIP	Control (no sugar)	0	1.67 ± 0.01 ^c^	0.55 ± 0.01 ^c^	0.99
	Sucrose	10	1.65 ± 0.04 ^c^	0.39 ± 0.01 ^g^	0.99
		20	1.08 ± 0.01 ^g^	0.57 ± 0.00 ^b^	0.99
	Lactose	10	1.35 ± 0.01 ^d^	0.56 ± 0.00 ^c^	0.99
		20	1.11 ± 0.01 ^f^	0.59 ± 0.01 ^a^	0.99
	Xylitol	10	2.22 ± 0.02 ^a^	0.42 ± 0.00 ^f^	0.99
		20	1.28 ± 0.01 ^e^	0.53 ± 0.01 ^d^	0.99
	Sorbitol	10	1.72 ± 0.01 ^b^	0.47 ± 0.02 ^e^	0.99
		20	1.35 ± 0.06 ^d^	0.51 ± 0.01 ^e^	0.99

The values represent the means of triplicate measurements ± SD. Means with different lowercase letters ^a–g^ within each column at each binder type are significantly different (*p* < 0.05).

**Table 5:** Values of dynamic rheological parameters at 6.28 rad·s^−1^ of raw and agglomerated MPIPs prepared with various sugar binders at different concentrations.

Sample	Binder Type	Binder Conc. (%)	*G*′ (Pa)	*G*′′ (Pa)	tan *δ*
Raw MPIP			1.24 ± 0.08 ^i^	3.17 ± 0.19 ^i^	2.57 ± 0.16 ^a^
Agglomerated MPIP	Control (no sugar)	0	3.31 ± 0.20 ^e^	4.27 ± 0.17 ^e^	1.29 ± 0.03 ^e^
	Sucrose	10	3.78 ± 0.27 ^d^	5.21 ± 0.28 ^d^	1.38 ± 0.03 ^d^
		20	2.59 ± 0.10 ^f^	3.95 ± 0.09 ^f^	1.53 ± 0.02 ^c^
	Lactose	10	2.58 ± 0.13 ^f^	3.89 ± 0.11 ^f^	1.51 ± 0.04 ^c^
		20	2.06 ± 0.03 ^h^	3.65 ± 0.07 ^g^	1.77 ± 0.02 ^b^
	Xylitol	10	7.12 ± 0.30 ^a^	6.56 ± 0.25 ^a^	0.92 ± 0.03 ^g^
		20	2.26 ± 0.26 ^g^	3.45 ± 0.17 ^h^	1.54 ± 0.10 ^c^
	Sorbitol	10	6.54 ± 0.10 ^b^	6.26 ± 0.19 ^b^	0.96 ± 0.04 ^g^
		20	5.08 ± 0.22 ^c^	6.04 ± 0.22 ^c^	1.19 ± 0.10 ^f^

The values represent the means of triplicate measurements ± SD. Means with different lowercase letters ^a–i^ within each column at each binder type are significantly different (*p* < 0.05).

## Data Availability

All the results showed in the manuscript could be requested to the corresponding author who would provide them.
